# Serum calcium, alkaline phosphotase as risk factors for bone metabolism in Alzheimer's disease

**DOI:** 10.1002/alz.093518

**Published:** 2025-01-09

**Authors:** Rupesh RSV Chintamanibatla, Preet Mehta, Sagar Kamarthi, Vidyani Suryadevara

**Affiliations:** ^1^ Northeastern University, Boston, MA USA; ^2^ Stanford University, Stanford, CA USA

## Abstract

**Background:**

Alzheimer's disease (AD) is a neurodegenerative disorder characterised by cognitive decline and progressive deterioration of brain function. Recent research has suggested a complex interplay between AD and bone health, with individuals affected by AD exhibiting an increased propensity for fractures and falls. Our preclinical studies in PSEN, MAPT P301 S and FDD mice have shown sex‐dependent changes in the bone in AD mice, compared to their age‐matched wild type mice. To delve deeper into the molecular underpinnings of this relationship, our study leverages data from the Alzheimer's Disease Neuroimaging Initiative (ADNI) database to investigate alterations in bone‐specific markers during AD pathology; to unravel potential links between bone metabolism and AD progression.

**Methods:**

Data from ADNI‐1(n=800), ADNI‐GO (n=700), ADNI‐2 (n=1300) and ADNI‐3 (n=2000) was considered for analysis. Spearman correlation analysis was performed between bone markers i.e. calcium and alkaline phosphatase with AD pathology‐ imaging and cognitive assessment.

**Results:**

Calcium levels and alkaline phosphate levels which are critical for bone metabolism were found to be significantly lower in patients with EMCI, LMCI and AD compared to control patients. A negative correlation was found between Calcium levels and Left Hippocampal Volume (Corr Coeff = ‐0.025, p = 1.38e‐19), Right Hippocampal Volume (Corr Coeff = ‐0.030, p = 1.95e‐26), and Montreal Cognitive Assessment (MOCA) scores (Corr Coeff = ‐0.015, p = 4.34e‐08). Alkaline Phosphatase levels show a strong negative correlation with Left Hippocampal Volume (Corr Coeff = ‐0.075, p = 8.45e‐154) and Right Hippocampal Volume (Corr Coeff = ‐0.078, p = 5.74e‐168) and a negative correlation with MOCA scores (Corr Coeff = ‐0.065, p = 9.02e‐118). The negative correlations between Calcium and Alkaline Phosphatase levels with left and right hippocampal volume suggest that lower bone‐specific markers are associated with reduced hippocampal volume, elucidating brain‐bone crosstalk during AD

**Conclusion:**

The study reveals correlation between serum bone markers (calcium, alkaline phosphatase) and declining hippocampal volume and cognitive function in Alzheimer's, suggesting a potential link between bone health and AD pathology. This discovery paves the way for deeper understanding and new avenues for diagnosis and treatment.

